# Use of Multiple Tobacco and Tobacco-Like Products Including Heated Tobacco and E-Cigarettes in Japan: A Cross-Sectional Assessment of the 2017 JASTIS Study

**DOI:** 10.3390/ijerph17062161

**Published:** 2020-03-24

**Authors:** Takefumi Sugiyama, Takahiro Tabuchi

**Affiliations:** 1 Osaka University School of Medicine, Suita city, Osaka 565-0871, Japan; sugiyama.tkfm@gmail.com; 2 Cancer Control Center, Osaka International Cancer Institute, 1-69, Ohtemae 3-chome, Chuo-ku, Osaka 541-8567, Japan

**Keywords:** multiple tobacco products use, cigarettes, heated tobacco products, electronic cigarettes, prevalence, Japan

## Abstract

Information on the use of multiple tobacco and tobacco-like products (hereafter multiple tobacco products use, i.e., use of more than one product) is important for tobacco control. Use of heated tobacco products (HTPs), which first became popular in Japan, has been spreading over the world, while information about use of multiple tobacco products, including HTPs, is insufficient. We analyzed data of 10,114 responders from the 2017 Japan “Society and New Tobacco” Internet Survey (JASTIS) study. The prevalence and adjusted odds ratio (aOR) of multiple tobacco products use were estimated with inverse probability weighting using multivariable logistic regression models to approximate the results to whole Japanese estimates. Tobacco and tobacco-like products included cigarettes, cigars, e-cigarettes, HTPs, pipes/water pipes, and smokeless tobacco products. Among Japanese adults, 18.4% were single tobacco product users and 3.2% were multiple tobacco product users in 2017. Among current product users (100%), cigarettes were the most popular product in single (78.8%) and multiple (14.2%) tobacco products use, while HTPs were the second most popular product in single (5.2%) and multiple (10.6%) tobacco products use. People with no perception of risk regarding e-cigarettes/HTPs were more likely to use multiple tobacco products (aOR = 1.47, 95% CI = 1.12–1.92) than those who perceived a risk. Prevalence rates and predictors of multiple tobacco products use, including HTPs, were studied first. In multiple tobacco products use, high popularity of HTPs among current product users was revealed. Risk perception of e-cigarettes/HTPs was associated with multiple tobacco products use. This study provides baseline information on multiple tobacco products use in Japan, which will enable the examination of trends in the future.

## 1. Introduction

Cigarette smoking is a major cause of preventable diseases but remains popular throughout the world, despite the decline in prevalence in many countries [1]. At the same time, novel tobacco and tobacco-like products, such as heated tobacco products (HTPs) and electronic cigarettes (e-cigarettes), have been aggressively marketed [2,3,4]. With the increasing number of new products available to the public, the landscape of tobacco and tobacco-like products use has changed dramatically. For example, e-cigarettes have become increasingly popular in the US while the prevalence of cigarette smoking is decreasing [5]. 

The use of multiple tobacco and tobacco-like products (hereafter multiple tobacco products use, i.e., use of more than one product) has become a major concern. In the US, approximately 40% of youth and adult tobacco users used multiple tobacco products in 2013-2014 [6]. Despite the expectancy of harm-reduction efficacy of novel products, previous studies have reported some negative effects (e.g., gateway) on adolescents as well as adults: e-cigarette use was associated with cigarette smoking among adolescents [7,8], HTP use among current cigarette users was not relevant to an intention to cease smoking [9], and multiple tobacco products use potentially increased the risk of problematic behavior, such as binge drinking [10]. Although HTP use is spreading, potential predictors of multiple tobacco products use, including HTPs, are not well understood.

Japan is the ninth highest consumer of cigarettes in the world [11], and is at the forefront of novel tobacco product sales. The HTPs Ploom TECH, IQOS, and glo were introduced into the Japanese market in the early days of their existence. The effect of HTPs on the decline in cigarette sales has been reported previously [12]. In Japan, HTPs are regulated just like cigarettes under the Tobacco Industries Act, because some components of HTPs are made from tobacco leaves. However, the sale of nicotine e-cigarettes has been banned under the Pharmaceutical Affairs Act since 2010, although personal purchase through the Internet is still permitted. As for smokeless tobaccos, Japan Tobacco (JT) has been selling “Zero style” (currently “Zero style stix”), which is dry snuff to be orally inhaled, since 2010 and “Zero style snus”, which is chewing tobacco comprising a sachet (also called a portion) filled with tobacco leaves, since 2013.

Recently, novel tobacco products have been developing rapidly, so studies about multiple tobacco products use are soon outdated. The objectives of the current study are (1) to estimate the prevalence of multiple tobacco products use, including HTPs and e-cigarettes; and (2) to explore predictors of multiple tobacco products use.

## 2. Materials and Methods 

### 2.1. Internet Survey

We used data from The Japan “Society and New Tobacco” Internet Survey (JASTIS): A longitudinal internet cohort study of HTPs, e-cigarettes, and conventional tobacco products in Japan [13]. Cross-sectional data from two cohorts of the 2017 JASTIS study were integrated and analyzed in this study. These cohorts comprised (1) respondents who had been followed up since the 2015 baseline survey: men and women aged 15–69 years in 2015 (n = 4217; response rate of 51.2% in 2017), and (2) respondents from a new baseline survey in 2017: men and women aged 15–69 years in 2017 (n = 5897; this survey was closed when the target number of respondents who had answered all the questionnaire items was reached). The baseline participants were randomly selected from panel members. The follow-up survey for cohort (1) was conducted from 27 January to 27 February 2017 and the baseline survey for cohort (2) was from 24 February to 13 March 2017. Further details are available in the cohort profile paper [13]. 

The survey panel was managed by a major, nationwide, internet research agency, Rakuten Research, which maintains a pool of 2.3 million panelists covering all social categories, such as education, housing tenure, and marital status, as defined by the Japanese census [14]. Members of the survey panel were initially recruited through the Rakuten agency group. At the time of registration, participants were required to provide information such as sex, age, occupation, and area of residence and to agree that they would participate in different research surveys with web-based written consent. Minors provided their consent with approval from their parents or guardians. After exclusion of those whose responses showed unnatural discrepancies (e.g., respondents who chose the same number throughout a set of questions), 10,114 respondents (4,217 from cohort (1) and 5,897 from cohort (2)) were analyzed using inverse probability weighting (IPW) for being a respondent in an internet survey to approximate a nationally representative sample [15,16]. There were no missing data in any factors used in the analysis.

### 2.2. Measures

#### 2.2.1. Current Tobacco and Tobacco-Like Products Use

Current tobacco and tobacco-like products use was determined by asking: “On how many days have you used the following products in the previous 30 days?” The options were “factory-made cigarettes”, “roll-your-own cigarettes”, “Ploom TECH”, “IQOS”, “glo”, “nicotine e-cigarette”, “non-nicotine e-cigarettes”, “e-cigarettes with unknown nicotine content”, “cigars”, “pipe”, “kiseru (Japanese pipe)”, “chewing tobacco”, “snuff”, and “water pipe (hookah)”. A current tobacco product user was defined as a person who had used at least one product in the previous 30 days. A current cigarette user was defined as a person who had smoked a cigarette (factory-made or roll-your-own) on at least one day in the previous 30 days. Similarly, a current HTP user was defined as a person who had used Ploom TECH, IQOS, or glo on at least one day in the previous 30 days; a current e-cigarette user was defined as a person who had used nicotine e-cigarettes, non-nicotine e-cigarettes, or e-cigarettes with unknown nicotine content; a current pipe/water pipe tobacco user was defined as a person who used a pipe, kiseru, or water pipe; a current smokeless tobacco user was defined as a person who used chewing tobacco or snuff. Among current product users, those who currently used one product exclusively were categorized as single product users with respect to each product measured. Multiple product users included dual product users (people who currently used two products) and poly product users (people who currently used three or more products).

#### 2.2.2. Risk Perception of Cigarettes

Respondents were asked the question: “Do you think that smoking cigarettes causes lung cancer?” The options were “definitely yes”, “probably yes”, “probably no”, and “definitely no”. Those who answered “definitely yes” or “probably yes” were defined as perceiving risk associated with smoking cigarettes.

#### 2.2.3. Risk Perception of E-cigarettes and HTPs

Respondents were asked the question: “Do you think e-cigarettes/HTPs are not harmful for users?” The options were “definitely yes”, “probably yes”, “probably no”, “definitely no”, and “I do not know these products”. Respondents who answered “definitely no” or “probably no” were defined as perceiving risk associated with use of e-cigarettes/HTPs.

#### 2.2.4. Other Variables

Other variables measured were age (15–24, 25–34, 35–44, 45–54, 55–64, 65–71), sex, marital status (married, never married, widowed/divorced), education (junior high school, high school, technical school or junior college, university (4 years or more)), self-rated health (good, middle, poor), and workplace indoor smoking ban status (no ban including smoking room/corner, complete ban, not working/do not know).

### 2.3. Statistical Analysis

As internet survey respondents are not representative of the general population, we conducted statistical adjustment to account for bias. Harmonization of the data with a major national and representative cross-sectional study (i.e., the Comprehensive Survey of Living Conditions of People on Health and Welfare (CSLCPHW), conducted by the Japanese Ministry of Health, Labor and Welfare) allowed us to pool data, providing the potential capacity to adjust for being a respondent in an internet survey [15,17]. Using this method, we could approximate our estimate to a nationally representative estimate with inverse probability weighting to account for baseline characteristics such as socio-demographic, health-related, and tobacco-related factors [15,17]. Details have also been given in a previous report [15]. The efficacy of adjustment using IPW is shown in Table S1.

We used data from our internet surveys and CSLCPHW for a multivariable logistic regression model and calculated the propensity score (PS) for each of 12 strata (sex × age groups), which set a deductive sample size in the internet surveys. For males and females aged 20–71 years, we adjusted residence, marital status, education, household, occupation, self-rated health, and smoking status for the 2016 CSLCPHW. For males and females aged 15–19 years, we adjusted residence, education, household, self-rated health, and smoking status for the 2010 CSLCPHW, because data for smoking status in minors were not available in the 2016 CSLCPHW.

To estimate the prevalence of multiple tobacco products use in Japan, weighted percentages were estimated with inverse probability weighting (IPW) adjustments and 95% confidence intervals (CIs) for single and multiple tobacco products use (defined above) using Wald and exact methods. To explore predictors of multiple tobacco products use, we estimated adjusted odds ratios (aORs) and 95% confidence intervals (CIs) for multiple tobacco products use by multivariable logistic regression models using standardized IPW, adjusting for tobacco-related, sociodemographic, and other factors. The survey data were selected and the IPW was calculated using SAS V.9.3.; the prevalence and predictors of multiple tobacco products use were estimated with R V.3.3.3. using the following library: DescTools for calculation of 95% confidence intervals [18]. The study was reviewed and approved by the Research Ethics Committee of the Osaka International Cancer Institute (no. 1412175183).

## 3. Results

### 3.1. Prevalence of Current Product Use

The prevalence of total, single, and multiple current products use is shown in Table 1. Among Japanese adults, 21.6% were current product users; 20.1% currently used cigarettes; 18.4% currently used a single product; 17.0% currently used cigarettes only; 1.1% currently used HTPs only; and 0.2% currently used e-cigarettes only. In terms of multiple tobacco products use, 3.2% currently used multiple tobacco products; 2.6% currently used dual products; 2.5% currently used dual products including cigarettes; 1.6% currently used both cigarettes and HTPs; 0.6% currently used both cigarettes and e-cigarettes; and 0.6% currently used more than two products. The weighted percentage of smoking (20.1%) was close to the nationally representative estimate of smoking prevalence (19.8%) (CSLCPHW 2016) [19]. Unweighted results are shown in Table S2.

### 3.2. Percentage Use of Products

**Figure 1:**
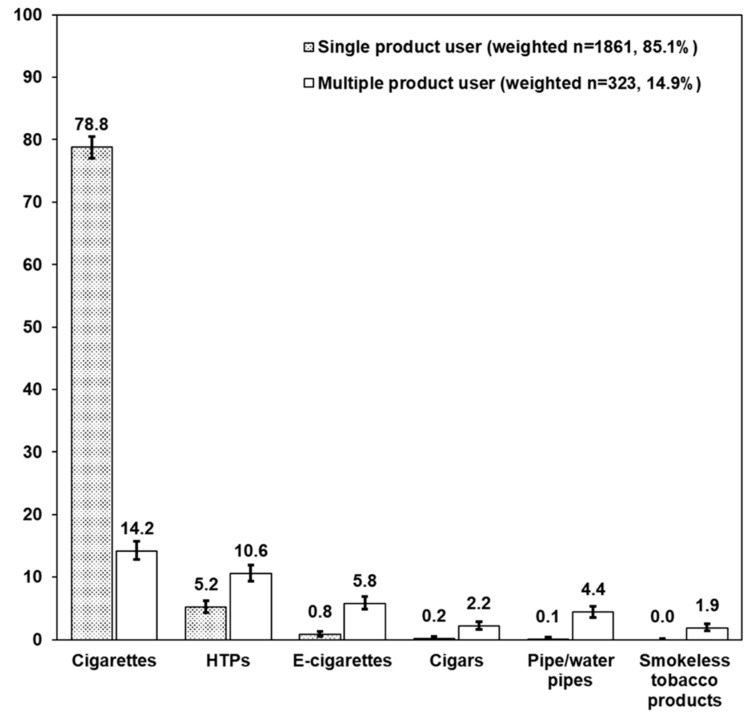
Percentages of tobacco and tobacco-like products use among current product users (weighted n = 2183, 100%). Error bars represent 95% confidence interval.

The percentage use of each product among current product users is shown in Figure 1. Cigarettes were the most popular among current product users for single (78.8%) and multiple (14.2%) products use, while HTPs were the second most popular for single (5.2%) and multiple (10.6%) products use. Products other than cigarettes were predominantly used by multiple tobacco product users.

### 3.3. Product Use Pattern

The percentage current use of each product according to characteristics is shown in Table 2. The prevalence of multiple tobacco products use was higher among males (5.3%) than females (1.0%). By age group, the prevalence of multiple tobacco products use was highest among adults aged 25 to 34 years (6.3%). By risk perception of cigarettes, the prevalence of multiple tobacco products use was higher among those who did not perceive any risk (6.1%) than those who did (2.8%). In the same way, by risk perception of e-cigarettes/HTPs, the prevalence of multiple tobacco products use was higher among those who did not perceive any risk (4.8%) than in those who did (3.5%). Unweighted results are shown in Table S3.

### 3.4. Predictors of Multiple Tobacco Products Use

Adjusted odds ratios (aORs) from the multivariable logistic regression analysis are shown in Table 3. The odds of multiple tobacco products use were significantly lower for widowed/divorced (aOR = 0.37, 95% CI = 0.19–0.69) than married, but higher for male (aOR = 1.55, 95% CI = 1.09–2.20) than female, adults aged 25 to 34 years (aOR = 2.17, 95% CI = 1.55–3.02) than aged 35 to 44 years, adults who were junior high school graduates (aOR = 1.73, 95% CI = 1.05–2.86) than university (4 years or more) graduates, and no workplace indoor smoking ban (aOR = 1.79, 95% CI = 1.12–2.87) than complete ban. Compared with those who perceived a risk in using e-cigarettes/HTPs, the odds of multiple tobacco products use were significantly higher for those who perceived no risk in using e-cigarettes/HTPs (aOR = 1.47, 95% CI = 1.12–1.92). Unweighted results are shown in Table S4. Results slightly differ between weighted and unweighted models, while risk perception of e-cigarettes/HTPs was found as a predictor of multiple tobacco products use in both models.

## 4. Discussion.

### 4.1. Prevalence of Current Product Use

To our knowledge, this is the first report on multiple tobacco products use in Japan, a country which currently has a more than 80% share in the worldwide market of IQOS, i.e., a major brand of HTPs. In the current study, we report the percentages of tobacco and tobacco-like products users in Japan in 2017 (Table 1): 21.6% of Japanese adults are current product users; 18.4% and 3.2% currently use single and multiple tobacco products, respectively. Conventional combustible cigarettes were still the most popular product in both single (17.0%) and multiple (3.1%) products use in Japan. This smoking rate is similar to the results from a nationally representative survey, the health and nutrition survey in Japan conducted by the Ministry of Health, Labor and Welfare (17.7%) in 2017 [20] as well as the 2016 CSLCPHW (19.8%) [19]. Figure 1 shows HTPs are used more frequently by multiple product users (10.6%) than single ones (5.2%). Total HTP users accounted for 15.8% of current product users. Although HTPs have come to the market recently, they are the second most popular tobacco product among current product users. The popularity of HTPs rather than e-cigarettes may be due to the difference in product regulation in Japan. The sale of nicotine-containing e-cigarettes has been banned in Japan (nicotine is treated as a pharmaceutical agent), while HTPs, that also contain nicotine, are not banned (they are treated as tobacco products). In contrast, 99.0% of e-cigarettes sold in the US in 2015 contained nicotine, but the sale of HTPs was not allowed at that time [21].

### 4.2. Predictors of Multiple Tobacco Products Use

Having no perception of risk related to e-cigarettes/HTPs had higher odds for multiple tobacco products use than did having a perception of risk, whereas risk perception of combustible cigarettes had no significant association with multiple tobacco products use. Strong adverse health effects of cigarettes have been studied and publicized for a long time [22,23,24] and this has contributed to the increase in risk perception of combustible cigarettes. However, there is far less information on the risk of HTPs compared with other products. Under these circumstances, tobacco companies promote HTPs as being far less harmful than conventional cigarettes although there is not sufficient evidence to reach an agreement about the health effects of these products [25,26,27]; this can lead to a low level of risk perception regarding e-cigarettes/HTPs. A previous study revealed that the packaging design of a novel tobacco product, IQOS, changed perception of product risk among current users [28]. By running campaigns that provide risk information based on the interests of the tobacco industry, tobacco companies may lower the perception of risk related to e-cigarettes/HTPs and encourage the use of these tobacco products.

Multiple tobacco products use was associated with male, age 25–34 years, and junior high school graduate (lowest education level), which was consistent with previous reports [8,29,30,31,32]. Low education level relates to both lower socioeconomic status and lower health awareness [33], which may result in the use of multiple tobacco products. Compared with a complete workplace indoor smoking ban, no ban was significantly associated with multiple tobacco products use. The contribution of workplace indoor smoking bans to the reduction of smoking prevalence has been reported previously [34]. Our study suggests that a complete workplace indoor smoking ban may be effective for the prevention of multiple tobacco products use. Moreover, multiple tobacco products use was negatively associated with widowed/divorced, and unawareness of e-cigarettes/HTPs compared with their counterpart categories. In terms of marital status, our results were different from a previous study in the US that showed a higher odds ratio for multiple tobacco products use among never married persons than married [30]. Although the reason for this difference is unknown, different preference for HTPs by characteristics may occur in this new era of HTP proliferation in Japan. The negative association between unawareness of e-cigarettes/HTPs and multiple tobacco products use was natural, but reflected the fact that the second and third most popular products, namely HTPs and e-cigarettes, have become major products in multiple tobacco products use.

### 4.3. Study Limitations

This study has several limitations. First, since it was a cross-sectional study, our results could not indicate causality, only association between variables. Second, our study was based on an internet survey and, thus, we used IPW adjustment to account for being an internet survey respondent using nationally representative data. Since this method cannot completely adjust for the difference in respondents between an internet survey and a nationwide representative survey, the problem of generalizability remains. Third, we possibly failed to provide a validated estimate for non-popular products (e.g., smokeless tobacco products), since the prevalence was very low and our sample size was limited.

## 5. Conclusions

This study provides baseline information on multiple tobacco products use (use of more than one product) in Japan, which will enable the examination of trends in the future. Multiple tobacco product users accounted for 3.2% of Japanese adults in 2017. Whereas the most popular product among multiple tobacco products use was combustible cigarettes, HTPs are the second most popular, and e-cigarettes (including nicotine e-cigarettes, non-nicotine e-cigarettes, and e-cigarettes with unknown nicotine content) the third. Risk perception of e-cigarettes/HTPs was associated with multiple tobacco products use. 

## Figures and Tables

**Table 1:** Prevalence of total, single, and multiple current products use * among participants in Japan (N = 10114).

Tobacco Product Use	N	Weighted N	Weighted % (95% CI)
Total Product Use (Tobacco and Tobacco-like Products Use)	1771	2183	21.6 (20.8–22.4)
Cigarette Use	1619	2030	20.1 (19.3–20.9)
Single Product Use	1526	1861	18.4 (17.6–19.2)
Cigarettes ^†^	1388	1720	17.0 (16.3–17.8)
HTPs ^‡^	112	115	1.1 (0.9–1.4)
E-cigarettes ^§^	15	18	0.2 (0.1–0.3)
Cigars	8	5	0.05 (0.02–0.11)
Pipe/Water Pipes	3	3	0.03 (0.00–0.08)
Smokeless Tobacco Products ^¶^	0	0	0.00 (0.00–0.04)
Multiple Products Use	245	323	3.2 (2.9–3.6)
Poly Products Use	58	62	0.6 (0.5–0.8)
Dual Products Use	187	260	2.6 (2.3–2.9)
Dual Products Use with Cigarettes	178	250	2.5 (2.2–2.8)
HTPs and Cigarettes	126	165	1.6 (1.4–1.9)
E-cigarettes and Cigarettes	34	64	0.6 (0.5–0.8)
Cigars and Cigarettes	8	6	0.1 (0.0–0.1)
Pipe/Water Pipes and Cigarettes	9	14	0.1 (0.1–0.2)
Smokeless Tobacco Products and Cigarettes	1	0	0.004 (0.000–0.044)
No Current Product Use	8343	7931	78.4 (77.6–79.2)

* Current product use was defined as use of a product even once in previous 30 days. ^†^ Cigarettes include factory-made and roll-your-own cigarettes. ^‡^ HTPs include Ploom TECH, IQOS, and glo. ^§^ E-cigarettes include e-cigarettes with/without nicotine, and e-cigarettes with unknown nicotine. ^¶^ Smokeless tobacco products include chewing tobaccos and snus. Abbreviations: HTPs, heated tobacco products; e-cigarettes, electronic cigarettes.

**Table 2:** Percentages of current product use status according to characteristics.

Characteristics	N (weighted N)	Product Use Status, weighted %				
		Single Product		Multiple Products		
		Any Product	Cigarettes	Multiple (≥2) *	Dual *	Poly (≥3) *
Overall	10114 (10114)	18.4 (17.6–19.2)	17.0 (16.3–17.8)	3.2 (2.9–3.6)	2.6 (2.3–2.9)	0.6 (0.5–0.8)
Sex						
Male	5142 (5142)	28.2 (27.0–29.4)	26.3 (25.1–27.5)	5.3 (4.7–6.0)	4.3 (3.7–4.8)	1.1 (0.8–1.3)
Female	4972 (4972)	8.3 (7.5–9.0)	7.4 (6.7–8.1)	1.0 (0.7–1.2)	0.8 (0.6–1.1)	0.2 (0.0–0.3)
Age group (years)						
15–24	1071 (1063)	7.6 (6.0–9.2)	6.6 (5.1–8.1)	1.4 (0.7–2.1)	1.1 (0.4–1.7)	0.3 (0.1–0.9)
25–34	1966 (1947)	17.5 (15.8–19.2)	14.6 (13.1–16.2)	6.3 (5.2–7.4)	4.9 (3.9–5.9)	1.4 (0.9–1.9)
35–44	2143 (2192)	21.2 (19.5–23.0)	19.3 (17.6–20.9)	3.5 (2.8–4.3)	2.4 (1.8–3.1)	1.1 (0.7–1.5)
45–54	2017 (2029)	21.6 (19.8–23.4)	20.5 (18.7–22.2)	2.9 (2.1–3.6)	2.7 (2.0–3.4)	0.2 (0.0–0.5)
55–64	1835 (1844)	21.0 (19.2–22.9)	20.7 (18.8–22.5)	2.2 (1.5–2.9)	2.0 (1.3–2.6)	0.2 (0.1–0.6)
65–71	1082 (1039)	14.2 (12.1–16.3)	14.1 (12.0–16.3)	1.0 (0.4–1.6)	0.9 (0.4–1.5)	0.01 (0.00–0.38)
Marital status						
Married	5791 (6387)	18.3 (17.3–19.2)	17.1 (16.1–18.0)	3.4 (2.9–3.8)	2.8 (2.4–3.2)	0.6 (0.4–0.8)
Never married	3622 (2993)	15.6 (14.3–16.9)	14.0 (12.8–15.3)	3.2 (2.6–3.9)	2.5 (1.9–3.0)	0.7 (0.4–1.0)
Widowed/divorced	701 (734)	30.8 (27.5–34.1)	28.8 (25.5–32.1)	1.5 (0.6–2.4)	1.4 (0.6–2.3)	0.1 (0.0–0.7)
Education						
Junior high school	278 (662)	26.5 (23.1–29.9)	25.1 (21.8–28.4)	4.6 (3.0–6.2)	4.6 (3.0–6.2)	0.04 (0.00–0.63)
High school	2819 (4515)	20.5 (19.3–21.7)	19.2 (18.0–20.3)	3.4 (2.9–3.9)	2.8 (2.4–3.3)	0.5 (0.3–0.7)
Technical school or junior college	2269 (2185)	13.3 (11.9–14.7)	12.1 (10.7–13.4)	2.5 (1.8–3.1)	1.9 (1.3–2.4)	0.6 (0.3–0.9)
University (4 years or more)	4748 (2752)	17.1 (15.6–18.5)	15.4 (14.0–16.7)	3.1 (2.5–3.8)	2.2 (1.7–2.8)	0.9 (0.5–1.3)
Self-rated health						
Good	5648 (3942)	18.0 (16.8–19.2)	16.5 (15.4–17.7)	3.3 (2.7–3.9)	2.7 (2.2–3.2)	0.6 (0.3–0.8)
Middle	3394 (5071)	19.4 (18.3–20.5)	18.0 (17.0–19.1)	3.2 (2.8–3.7)	2.6 (2.1–3.0)	0.7 (0.4–0.9)
Poor	1072 (1101)	15.2 (13.1–17.3)	14.0 (11.9–16.0)	2.6 (1.6–3.5)	2.1 (1.2–2.9)	0.5 (0.1–0.9)
Workplace indoor smoking ban status						
No ban (including smoking room/corner)	6594 (6271)	20.6 (19.6–21.6)	19.0 (18.0–20.0)	4.4 (3.9–4.9)	3.5 (3.1–4.0)	0.9 (0.6–1.1)
Complete ban	418 (592)	36.0 (32.1–39.8)	31.7 (27.9–35.4)	4.1 (2.5–5.7)	3.5 (2.0–5.0)	0.6 (0.1–1.6)
Not working/did not know	3102 (3251)	10.9 (9.9–12.0)	10.5 (9.4–11.5)	0.7 (0.4–1.0)	0.6 (0.3–0.8)	0.2 (0.0–0.3)
Risk perception of cigarettes						
Yes (perceived risk)	9121 (9027)	15.7 (15.0–16.5)	14.4 (13.7–15.1)	2.8 (2.5–3.2)	2.3 (2.0–2.6)	0.6 (0.4–0.7)
No (did not perceive risk)	993 (1087)	40.7 (37.7–43.6)	38.7 (35.8–41.6)	6.1 (4.7–7.5)	5.2 (3.9–6.5)	0.9 (0.3–1.4)
Risk perception of e-cigarettes/HTPs						
No (perceived risk)	6081 (5867)	19.5 (18.5–20.5)	18.1 (17.1–19.0)	3.5 (3.0–3.9)	2.7 (2.3–3.1)	0.7 (0.5–1.0)
Yes (did not perceive risk)	2077 (2232)	21.2 (19.5–22.8)	18.8 (17.2–20.5)	4.8 (3.9–5.7)	4.0 (3.2–4.9)	0.8 (0.4–1.1)
Did not know e-cigarettes/HTPs	1956 (2016)	12.1 (10.6–13.5)	12.0 (10.5–13.4)	0.7 (0.3–1.0)	0.5 (0.2–0.9)	0.1 (0.0–0.4)

* Dual, poly, and multiple products use were defined as current use of two, more than two, and more than one product(s), respectively.

**Table 3:** Predictors of multiple tobacco products use among current product users in Japan.

Variables	Adjusted ORs * (95% CI)	*P* ^†^
Sex		0.012
Male	1.55 (1.09–2.20)	
Female	1 (reference)	
Age group (years)		<0.001
15–24	1.16 (0.61–2.21)	
25–34	2.17 (1.55–3.02)	
35–44	1 (reference)	
45–54	0.83 (0.57–1.21)	
55–64	0.67 (0.44–1.03)	
65–71	0.65 (0.31–1.36)	
Marital status		0.002
Married	1 (reference)	
Never married	0.86 (0.64–1.16)	
Widowed/divorced	0.37 (0.19–0.69)	
Education		0.182
Junior high school	1.73 (1.05–2.86)	
High school	1.03 (0.76–1.39)	
Technical school or junior college	1.04 (0.70–1.54)	
University (4 years or more)	1 (reference)	
Self-rated health		0.371
Good	0.86 (0.66–1.13)	
Middle	1 (reference)	
Poor	1.16 (0.73–1.83)	
Workplace indoor smoking ban status		0.001
No ban (including smoking room/corner)	1.79 (1.12–2.87)	
Complete ban	1 (reference)	
Not working/did not know	0.83 (0.44–1.54)	
Risk perception of cigarettes		0.657
Yes (perceived risk)	1 (reference)	
No (did not perceive risk)	0.93 (0.68–1.27)	
Risk perception of e-cigarettes/HTPs		<0.001
No (perceived risk)	1 (reference)	
Yes (did not perceive risk)	1.47 (1.12–1.92)	
Did not know e-cigarettes/HTPs	0.43 (0.24–0.79)	

* Adjusted for all listed variables. ^†^ P for difference. Boldface indicates statistical significance of *p* < 0.05.

## Supplementary Materials

The following are available online at https://www.mdpi.com/1660-4601/17/6/2161/s1, Table S1: Percentages of current use of cigarettes, Table S2: Prevalence of total, single, and multiple current product use among participants in Japan (N = 10114) (unweighted results), Table S3: Percentages of current product use status according to characteristics (unweighted results), Table S4: Predictors of multiple tobacco products use among current product users in Japan (unweighted results).
