# Supplementary material for: Use of Multiple Tobacco and Tobacco-Like Products Including Heated Tobacco and E-Cigarettes in Japan: A Cross-Sectional Assessment of the 2017 JASTIS Study
Source: Int J Environ Res Public Health. 2020 Mar 24;17(6):2161. doi: 10.3390/ijerph17062161 (PMC7143444; doi:10.3390/ijerph17062161)
Supplement: Supplementary file 1 [file ijerph-17-02161-s001.pdf]

Supplementary data (online only)

Title

Multiple use of tobacco and tobacco-like products including heated tobacco and e-cigarettes in Japan: a cross-sectional assessment of the 2017 JASTIS study

Authors

Takefumi Sugiyama[1]; Takahiro Tabuchi, MD, PhD[2*]

List

Table S1. Percentages of current use of cigarettes
Table S2. Prevalence of total, single and multiple current product use among participants in Japan (N=10114) (unweighted results)
Table S3. Percentages of current product use status according to characteristics (unweighted results)
Table S4. Predictors of multiple product use among current product users in Japan. (unweighted results)

Table S1. Percentages of current use of cigarettes*

Age group (years)	Current cigarette use (%)		
	Unadjusted	Adjusted	Population-based survey†
Both sexes	16.0	20.1	23.4‡
Male			
Total	22.4	31.5	31.1
15-19	2.5	2.1	3.8
20-29	14.5	24.1	31.1
30-39	22.8	34.0	39.9
40-49	26.4	35.8	39.4
50-59	29.8	34.0	37.1
60-71	20.1	32.3	30.0§
Female			
Total	9.4	8.2	9.5
15-19	1.2	1.5	1.4
20-29	6.6	7.5	10.2
30-39	9.2	9.8	12.9
40-49	14.0	10.7	14.7
50-59	11.2	7.8	12.7
60-71	6.7	6.1	8.2§

*Cigarettes include factory-made and roll-your-own cigarettes. †Percentages of current smokers by age groups from a population-based survey, i.e. the CSLCPHW: in both sexes, figures for 15–19 years were from 2010 CSLCPHW and those for other age categories were from 2016 CSLCPHW. (data were available from the e-stat. URL: https://www.e-stat.go.jp/) ‡for 20-69 years. §for 60-69 years. CSLCPHW = Comprehensive Survey of Living Conditions of People on Health and Welfare.

Table S2. Prevalence of total, single and multiple current product use* among participants in Japan (N=10114) (unweighted results)

Tobacco product use	N	% (95% CI)
Total Product Use (Tobacco and Tobacco-like Product Use)	1771	17.5 (16.8–18.3)
Cigarette Use	1619	16.0 (15.3–16.7)
Single Product Use	1526	15.1 (14.4–15.8)
Cigarettes†	1388	13.7 (13.1–14.4)
HTPs‡	112	1.1 (0.9–1.3)
E-cigarettes§	15	0.1 (0.1–0.2)
Cigars	8	0.08 (0.03–0.16)
Pipe/Water Pipes	3	0.03 (0.01–0.09)
Smokeless Tobacco Products¶	0	0.00 (0.00–0.04)
Multiple Product Use	245	2.4 (2.1–2.7)
Poly Product Use	58	0.6 (0.4–0.7)
Dual Product Use	187	1.8 (1.6–2.1)
Dual Product Use with Cigarettes	178	1.8 (1.5–2.0)
HTPs and Cigarettes	126	1.2 (1.0–1.5)
E-cigarettes and Cigarettes	34	0.3 (0.2–0.5)
Cigars and Cigarettes	8	0.1 (0.0–0.2)
Pipe/Water Pipes and Cigarettes	9	0.09 (0.04–0.17)
Smokeless Tobacco Products and Cigarettes	1	0.01 (0.00–0.06)
No Current Product Use	8343	82.5 (81.7–83.2)

*Current product use was defined as 'use of a product even once in previous 30 days'.

†Cigarettes include factory-made and roll-your-own cigarettes.

‡HTPs include Ploom TECH, IQOS, and glo.

§E-cigarettes include e-cigarettes with/without nicotine, and e-cigarettes with unknown nicotine.

¶Smokeless tobacco products include chewing tobaccos and snus.

Abbreviations: HTPs, heated tobacco products; e-cigarettes, electronic cigarettes

Table S3. Percentages of current product use status according to characteristics (unweighted results)

Characteristics	N	Product Use Status, unweighted %				
		Single product		Multiple product		
		Any product	Cigarettes	Multiple(>=2)*	Dual*	Poly(>=3)*
**Overall**	10114	15.1 (14.4–15.8)	13.7 (13.1–14.4)	2.4 (2.1–2.7)	1.8 (1.6–2.1)	0.6 (0.4–0.7)
Sex						
Male	5142	20.6 (19.5–21.7)	18.8 (17.7–19.9)	3.8 (3.3–4.4)	2.9 (2.4–3.4)	1.0 (0.7–1.3)
Female	4972	9.4 (8.6–10.2)	8.5 (7.7–9.3)	1.0 (0.7–1.3)	0.8 (0.6–1.1)	0.2 (0.1–0.3)
Age group (years)						
15–24	1071	5.2 (4.0–6.7)	4.3 (3.2–5.7)	1.6 (0.9–2.5)	0.9 (0.4–1.7)	0.7 (0.3–1.3)
25–34	1966	13.2 (11.8–14.8)	10.8 (9.5–12.3)	3.5 (2.7–4.4)	2.4 (1.8–3.2)	1.0 (0.6–1.6)
35–44	2143	17.5 (15.9–19.2)	15.4 (13.9–17.0)	3.0 (2.3–3.8)	2.2 (1.6–2.9)	0.8 (0.5–1.3)
45–54	2017	19.7 (18.0–21.5)	18.3 (16.7–20.1)	2.7 (2.0–3.5)	2.3 (1.7–3.0)	0.4 (0.2–0.8)
55–64	1835	17.1 (15.4–18.9)	16.6 (14.9–18.4)	1.6 (1.1–2.3)	1.4 (0.9–2.0)	0.3 (0.1–0.6)
65–71	1082	11.6 (9.7–13.6)	11.4 (9.5–13.4)	1.1 (0.6–1.9)	1.0 (0.5–1.8)	0.1 (0.0–0.5)
Marital status						
Married	5791	15.3 (14.4–16.3)	14.0 (13.1–14.9)	2.3 (2.0–2.8)	1.8 (1.5–2.2)	0.6 (0.4–0.8)
Never married	3622	13.2 (12.1–14.4)	11.8 (10.8–12.9)	2.6 (2.1–3.2)	1.9 (1.5–2.4)	0.7 (0.4–1.0)
Widowed/divorced	701	23.0 (19.9–26.3)	21.5 (18.6–24.8)	2.3 (1.3–3.7)	2.1 (1.2–3.5)	0.1 (0.0–0.8)
Education						
Junior high school	278	20.9 (16.2–26.1)	17.6 (13.3–22.6)	4.3 (2.3–7.4)	4.0 (2.0–7.0)	0.4 (0.0–2.0)
High school	2819	17.5 (16.1–19.0)	16.2 (14.9–17.7)	2.7 (2.1–3.4)	2.3 (1.8–3.0)	0.4 (0.2–0.7)
Technical school or junior college	2269	13.9 (12.5–15.4)	12.5 (11.2–13.9)	1.3 (0.9–1.9)	0.9 (0.6–1.4)	0.4 (0.2–0.8)
University (4 years or more)	4748	13.9 (12.9–14.9)	12.6 (11.6–13.6)	2.7 (2.2–3.2)	1.9 (1.5–2.3)	0.8 (0.6–1.1)
Self-rated health						

Good	5648	14.2 (13.3–15.1)	12.9 (12.0–13.8)	2.4 (2.0–2.8)	1.8 (1.5–2.2)	0.6 (0.4–0.8)
Middle	3394	17.3 (16.0–18.6)	15.8 (14.6–17.1)	2.4 (1.9–3.0)	1.9 (1.5–2.4)	0.5 (0.3–0.8)
Poor	1072	12.8 (10.8–14.9)	11.4 (9.5–13.4)	2.9 (2.0–4.1)	2.1 (1.3–3.1)	0.8 (0.4–1.6)
Workplace indoor smoking ban status						
No ban (including smoking room/corner)	6594	17.0 (16.1–17.9)	15.3 (14.5–16.2)	3.0 (2.6–3.5)	2.4 (2.0–2.7)	0.7 (0.5–0.9)
Complete ban	418	30.9 (26.5–35.5)	26.3 (22.2–30.8)	5.0 (3.1–7.6)	3.8 (2.2–6.1)	1.2 (0.4–2.8)
Not working/did not know	3102	9.0 (8.0–10.0)	8.6 (7.7–9.7)	0.8 (0.5–1.1)	0.5 (0.3–0.8)	0.3 (0.1–0.5)
Risk perception of cigarettes						
Yes (perceived risk)	9121	12.6 (11.9–13.3)	11.3 (10.6–11.9)	2.1 (1.9–2.5)	1.6 (1.4–1.9)	0.5 (0.4–0.7)
No (did not perceive risk)	993	38.4 (35.3–41.5)	36.4 (33.4–39.4)	5.0 (3.8–6.6)	3.7 (2.6–5.1)	1.3 (0.7–2.2)
Risk perception of e-cigarettes/HTPs						
No (perceived risk)	6081	16.1 (15.2–17.1)	14.6 (13.8–15.5)	2.5 (2.2–3.0)	1.9 (1.6–2.3)	0.7 (0.5–0.9)
Yes (did not perceive risk)	2077	17.2 (15.6–18.9)	15.1 (13.6–16.7)	3.9 (3.1–4.8)	3.2 (2.5–4.0)	0.7 (0.4–1.1)
Did not know e-cigarettes/HTPs	1956	9.6 (8.3–11.0)	9.4 (8.1–10.8)	0.5 (0.2–0.9)	0.3 (0.1–0.7)	0.2 (0.1–0.5)

*Dual, poly and multiple product use were defined as current use of two, more than two and more than one tobacco product(s), respectively.

Table S4. Predictors of multiple product use among current product users in Japan (unweighted results)

**Variables**	**Adjusted ORs* (95% CI)**	***P*** [†]
Sex		0.003
Male	**1.69 (1.18–2.43)**	
Female	1 (reference)	
Age group (years)		0.001
15–24	1.81 (0.97–3.41)	
25–34	1.42 (0.97–2.10)	
35–44	1 (reference)	
45–54	0.76 (0.51–1.13)	
55–64	**0.56 (0.35–0.90)**	
65–71	0.67 (0.33–1.35)	
Marital status		0.795
Married	1 (reference)	
Never married	0.98 (0.71–1.35)	
Widowed/divorced	0.83 (0.47–1.45)	
Education		0.056
Junior high school	1.06 (0.53–2.12)	
High school	0.88 (0.64–1.21)	
Technical school or junior college	**0.56 (0.36–0.87)**	
University (4 years or more)	1 (reference)	
Self-rated health		0.052
Good	1.16 (0.85–1.57)	
Middle	1 (reference)	
Poor	**1.82 (1.14–2.92)**	
Workplace indoor smoking ban status		0.270
No ban (including smoking room/corner)	0.99 (0.60–1.64)	
Complete ban	1 (reference)	
Not working/did not know	0.67 (0.35–1.29)	
Risk perception of cigarettes		0.409
Yes (perceived risk)	1 (reference)	
No (did not perceive risk)	0.87 (0.61–1.22)	
Risk perception of e-cigarettes/HTPs		<0.001
No (perceived risk)	1 (reference)	
Yes (did not perceive risk)	**1.58 (1.16–2.15)**	
Did not know e-cigarettes/HTPs	**0.43 (0.22–0.85)**	

*Adjusted for all listed variables. [†] P for difference
